# Supplementary material for: CLAVATA signalling shapes barley inflorescence by controlling activity and determinacy of shoot meristem and rachilla
Source: Nat Commun. 2025 Apr 26;16:3937. doi: 10.1038/s41467-025-59330-z (PMC12033307; doi:10.1038/s41467-025-59330-z)
Supplement: Supplementary file 1 — Supplementary Information [file 41467_2025_59330_MOESM1_ESM.pdf]

CLAVATA signalling shapes barley inflorescence by controlling activity and determinacy of shoot meristem and rachilla

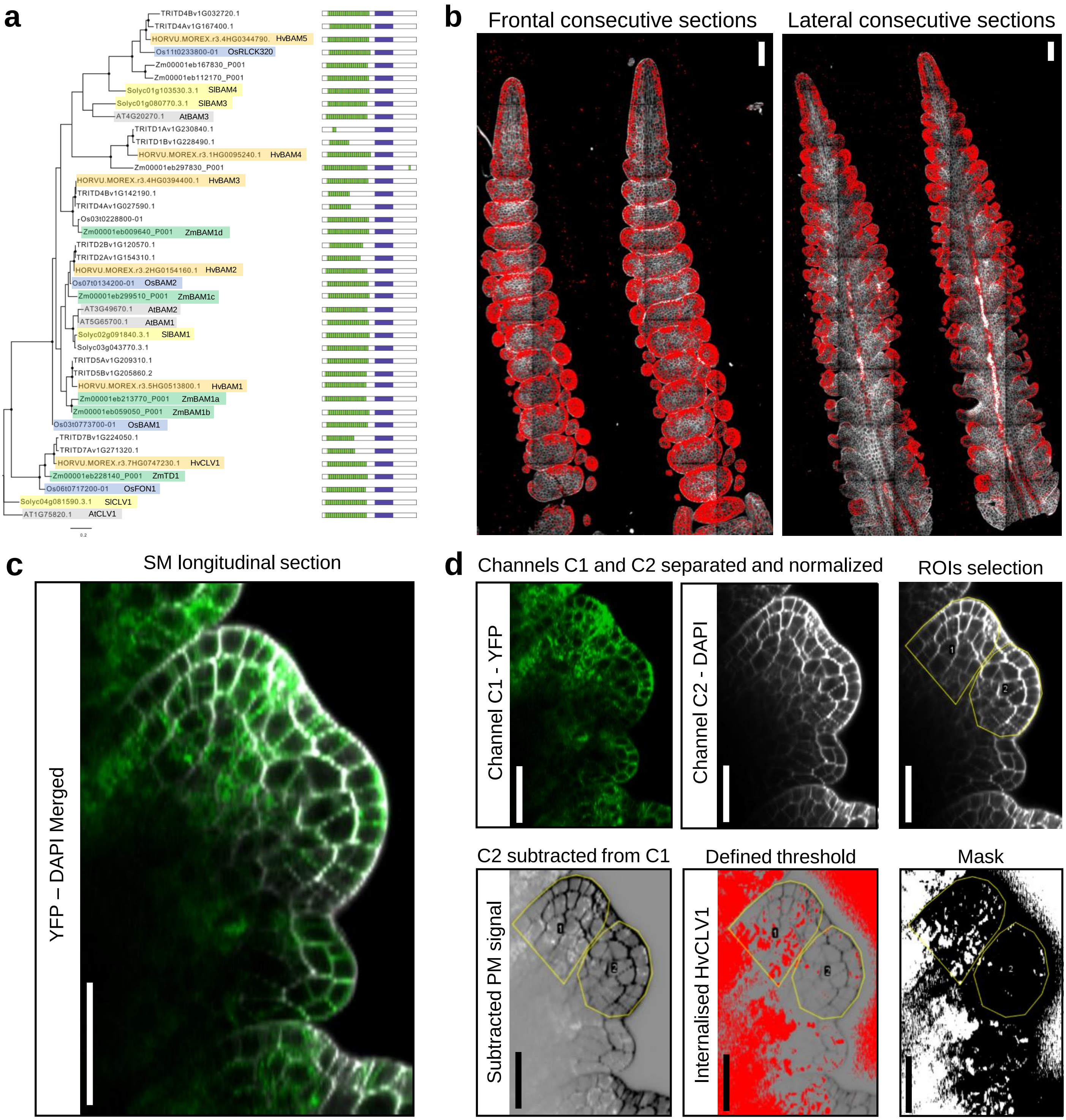

Supplementary Figure 1. **Phylogenetic analysis, expression pattern and quantification of HvCLV1 cytoplasmic internalisation**

a Maximum likelihood tree of the CLV1 clade. Dots indicate nodes with bootstrap values higher than 80. Genes identifiers and names of previously described genes are highlighted in different colours based on the species (*Arabidopsis thaliana* (grey), *Solanum lycopersicum* (yellow), *Zea mays* (green), *Oryza sativa japonica* (blue) and *Hordeum vulgare* (orange), together with a schematic representation of the predicted protein structure. Kinase domain as purple rectangle and LRRs as green rectangles. **b** Results from smRNA-FISH (Molecular Cartography^{TM}, Resolve Biosciences) experiment. The fluorescent signal in red shows the localisation of *HvCLV1* transcripts in frontal and lateral sections of the barley inflorescence at W3.5. Scalebar: 100 µm. **c** HvCLV1 protein localisation (green) in a spikelet longitudinal section. Scalebar: 50 µm. **d** Step-by-step illustration of the method used to quantify HvCLV1 internalisation in RP and FM using Fiji-software.

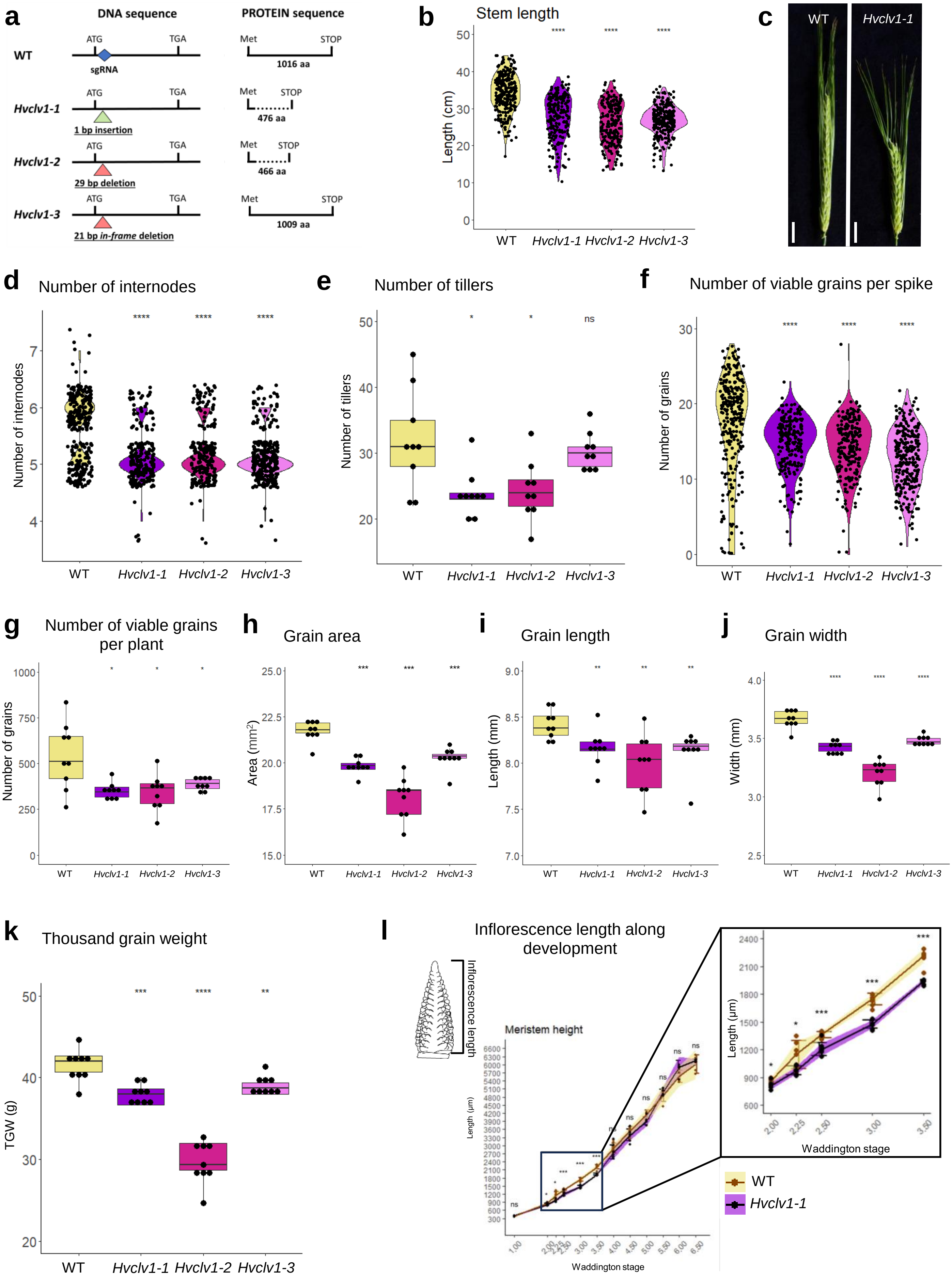
a
DNA sequence
PROTEIN sequence
WT
ATG
TGA
sgRNA
Met
STOP
1016 aa
Hvclv1-1
1 bp insertion
476 aa
Hvclv1-2
29 bp deletion
466 aa
Hvclv1-3
21 bp in-frame deletion
1009 aa
b
Stem length
Length (cm)
c
WT
Hvclv1-1
d
Number of internodes
e
Number of tillers
f
Number of viable grains per spike
Number of grains
g
Number of viable grains per plant
h
Grain area
Area (mm²)
i
Grain length
Length (mm)
j
Grain width
Width (mm)
k
Thousand grain weight
TGW (g)
l
Inflorescence length along development
Inflorescence length
Meristem height
Length (μm)
Waddington stage
WT
Hvclv1-1

Supplementary Figure 2. ***Hvclv1* mutant alleles plant and grain phenotypes**

a Schematic representation of the HvCLV1 DNA and protein sequence in WT and *Hvclv1-1, Hvclv1-2, Hvclv1-3* alleles. The blue square indicates the region targeted by the sgRNA, the triangles indicate the position of the selected mutation (insertions in green, deletions in red). The dotted lines indicate a different protein sequence to WT. **b-g** Plant and spike phenotype. Measurements of stem length (b), spike phenotype of WT and *Hvclv1-1* 60 DAS. Scale bar: 1.5 cm (c), number of internodes (d), number of tillers (e), number of viable grains per spike (f) and per plant (g). Dots indicate single measurement performed in each tiller from n=9 mature plants over n=3 independent experiments, and asterisks indicate the significant difference in comparison to WT using a two-sided Pairwise Wilcoxon rank sum test (b,d,f) and a two-sided Pairwise t-test (C,E). **h-k** Grain phenotype and weight: measurements of grain area (h), grain length (i), grain width (j) and Thousand grains weight (TGW) (k). Dots indicate the average value of measurements taken in n=150 mature grains from n=9 different plants. Asterisks indicate the significant difference in comparison to WT using a two-sided Pairwise Wilcoxon rank sum test (h,i) and a two-sided Pairwise t-test (j,k). **L** Inflorescence length of WT (yellow) and *Hvclv1-1* (purple). Measurements were taken from W1 to W6.5. Zoom-in plot between W2 and W3.5 on the right. n=10 over n=2 independent experiments. Dots represent single measurements; error bars represent standard deviation and the colored ribbons the interval of confidence. Asterisks indicate the significant difference to WT for each W using a two-sided Pairwise t-test. Boxplots: median (center line); upper and lower quartiles (box limits); 1.5x interquartile range (whiskers); points, outliers. Statistics: ns = non-significant (p-value > 0.05); * (p-value < 0.05); ** (p-value < 0.01); *** (p-value < 0.001); **** (p-value < 0.0001).

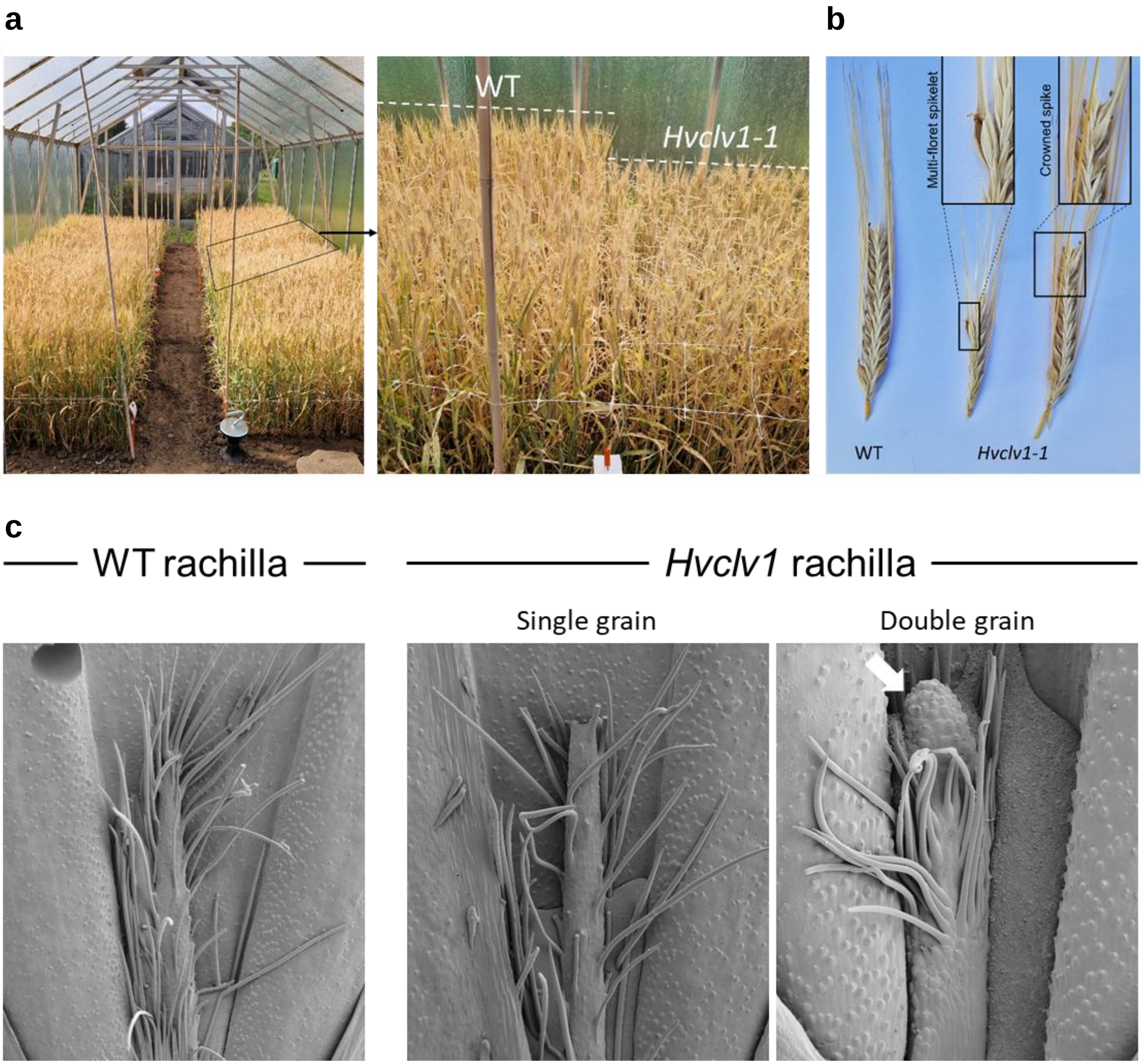

Supplementary Figure 3. ***Hvclv1-1* growing in semi-field conditions and mature rachilla phenotype**

a Pictures of the semi-field setup and zoom-in in WT and *Hvclv1-1* plants. In every plot, twelve rows of 44 grains were sown 11 cm apart (528 grains per 1.3mx1.35m =~300 grains per m^2) and grown between March and August 2023 in Germany. **b** Examples of spikes from WT and *Hvclv1-1* mutant showing multi-floret spikelets and crowned spikes. The average yield of two plots was 851.7 +- 85 grams for WT plants and 466.4 +- 20.4 grams for *Hvclv1-1.* Thousand Grain Weight (TGW) was 41.9 +- 0.1 g for WT plants and 38.0 +- 1.4 g for *Hvclv1-1* plants. **c** SEM pictures of mature rachilla from WT and *Hvclv1* single and double grains. The WT phenotype was observed in 9/9 samples, the *Hvclv1* rachilla showed a reduced number of hairs in 7/7 rachillae on single grains and an enlarged tip (white arrow) was observed in 1/3 double grains.

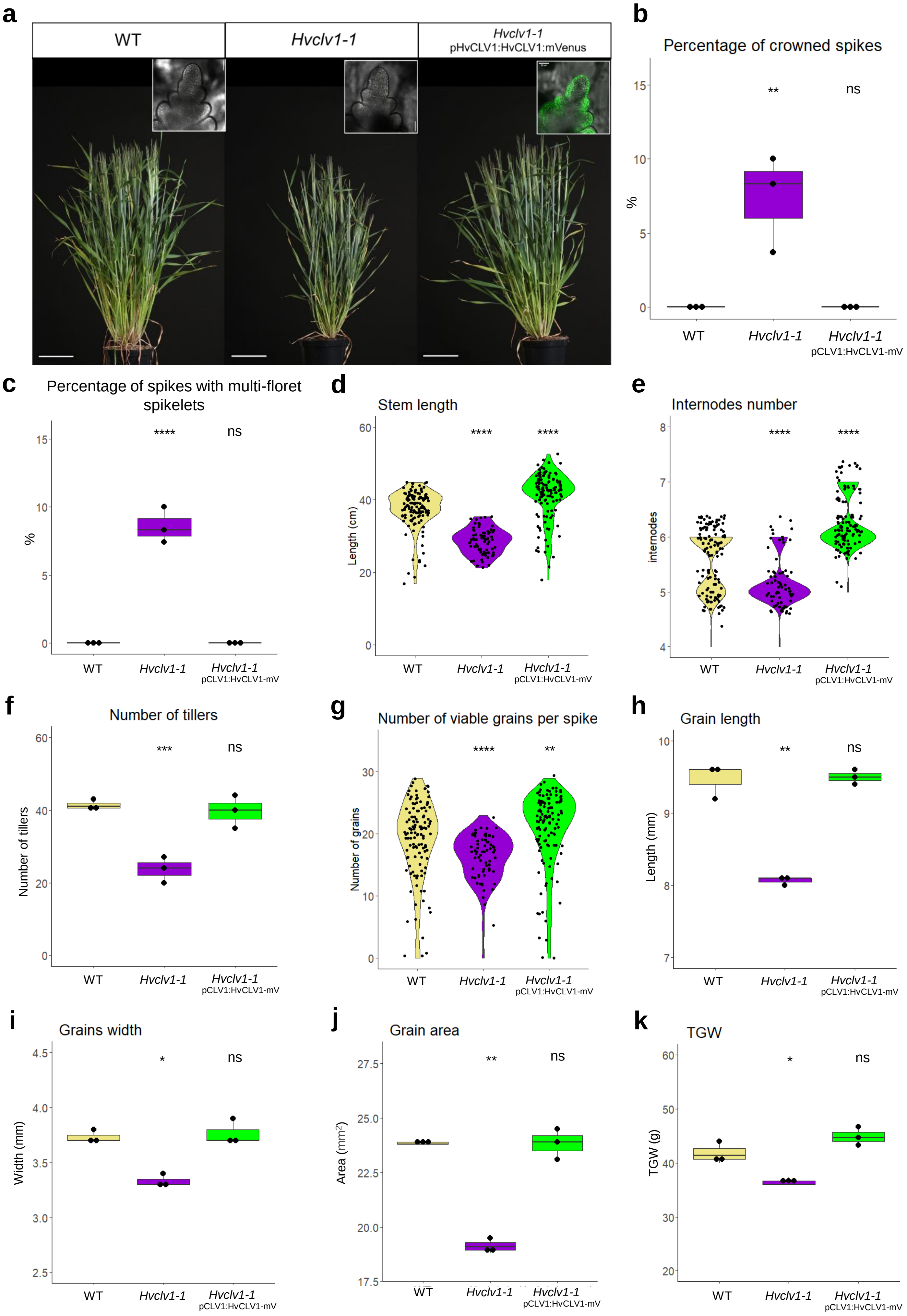
a
WT
Hvclv1-1
Hvclv1-1 pHvCLV1:HvCLV1:mVenus
b
Percentage of crowned spikes
c
Percentage of spikes with multi-floret spikelets
d
Stem length
Length (cm)
e
Internodes number
internodes
f
Number of tillers
g
Number of viable grains per spike
Number of grains
h
Grain length
Length (mm)
i
Grains width
Width (mm)
j
Grain area
Area (mm²)
k
TGW
TGW (g)
WT
Hvclv1-1
Hvclv1-1 pCLV1:HvCLV1-mV
ns

Supplementary Figure 4. **Plant and grain phenotypes of the *Hvclv1*/pCLV1:HvCLV1-mV complementation line**

a Images of WT and *Hvclv1-1* plant in comparison to *Hvclv1-1* carrying pHvCLV1:HvCLV1:mVenus plasmid (*Hvclv1/*pCLV1:HvCLV1-mV), pictures were taken 60 days after sowing. Each plant phenotype is combined with HvCLV1:mVenus expression (green) in the IM in the relative genetic background. Scale bar: 10 cm (plants), 50 µm (IMs). **b,c** Percentage of crowned spikes and spikes with multi-floret spikelets respectively in WT, *Hvclv1* and *Hvclv1/*pCLV1:HvCLV1-mV. Dots represent the percentage per plant and asterisks indicate the significant difference to WT using a two-sided Pairwise t-test. n=3. **d-g** Plant and spike phenotype. Measurements of stem length (d), number of internodes (e), number of tillers (f), number of viable grains per spike (g). Dots indicate single measurement performed in each tiller from three mature plants, and asterisks indicate the significant difference in comparison to WT using a two-sided Pairwise Wilcoxon rank sum test (d,e,g) and a two-sided Pairwise t-test (f). **h-k** Grain phenotype and weight: measurements of grain length (h), grain width (i), grain area (j) and Thousand grains weight (TGW) (k). Dots indicate the average of value of measurements taken in 150 mature grains from n=3 plants, asterisks indicate the significant difference in comparison to WT using a two-sided Pairwise Wilcoxon rank sum test. Boxplots: median (center line); upper and lower quartiles (box limits); 1.5x interquartile range (whiskers); points, outliers. Statistics: ns = non-significant (p-value > 0.05); * (p-value < 0.05); ** (p-value < 0.01); *** (p-value < 0.001); **** (p-value < 0.0001).

a Barley CLE peptides

Gene ID	Name	CLE domain sequence
HORVU.MOREX.r3.7HG0709200	HvCLE1	RLSPGGSNPQHH
HORVU.MOREX.r3.4HG0410450	HvCLE2	RLSPGGSNPQHH
HORVU.MOREX.r3.5HG0514970	HvCLE3	RLSPGGSNPQHH
HORVU.MOREX.r3.3HG0289600	HvCLE4	RLSPGGSDPQHH
HORVU.MOREX.r3.1HG0087130	HvCLE5	RTSPGGPDPQHH
HORVU.MOREX.r3.1HG0087050	HvCLE6	RKSPGGPDPQHH
HORVU.MOREX.r3.1HG0087060	HvCLE7	RKSPGGPDPQHH
HORVU.MOREX.r3.3HG0289520	HvCLE8	REVPGGPDPEHH
HORVU.MOREX.r2.7HG0588240	HvCLE9	REVPGGPDPIHH
HORVU.MOREX.r3.2HG0174890	HvFCP1	REVPTGPDPIHH
HORVU.MOREX.r3.4HG0360540	HvCLV3	RSVPAGPDPLHH
HORVU.MOREX.r3.1HG0008860	HvFOS1	RLVPTGPNPLHH
HORVU.MOREX.r3.1HG0007940	HvCLE10	RRVPTGPNPETP
HORVU.MOREX.r3.2HG0128960	HvCLE11	RRVPNSSDPLHN
HORVU.MOREX.r3.3HG0221960	HvCLE12	RPVPSCPDALHN
HORVU.MOREX.r3.1HG0076250	HvCLE13	RRIPKGPDPIHN
HORVU.MOREX.r3.4HG0335200	HvCLE14	RKVPNGPDPIHN
HORVU.MOREX.r2.5HG0433480	HvCLE15	RRVPNGPDPIHN
HORVU.MOREX.r3.2HG0107180	HvCLE16	RRVPNGPDPIHN
HORVU.MOREX.r3.2HG0202370	HvCLE17	RRVPTGPNPLHN
HORVU.MOREX.r3.7HG0728670	HvCLE18	RRIPTGPNPLHN
HORVU.MOREX.r3.6HG0559740	HvCLE19	RAVPTGANPLHN
HORVU.MOREX.r3.4HG0390560	HvCLE20	RPVPTGSNPLHN
HORVU.MOREX.r3.3HG0233620	HvCLE21	RMAPSGSNPLHN
HORVU.MOREX.r3.1HG0026860	HvCLE22	RFAPTGSNPLHN
HORVU.MOREX.r3.2HG0183900	HvCLE23	RLVPQGPNPLHN
HORVU.MOREX.r3.6HG0598450	HvCLE24	RMVPQGPNPLHN
HORVU.MOREX.r3.6HG0632110	HVCLE25	RAVPQGPNPLHN

b FCP1 orthologs - CLE domain

Gene ID	Name	CLE domain sequence
AT5G12990	AtCLE40	RQVPTGSDPIHH
HORVU.MOREX.r3.1HG0008860	HvFCP1	REVPTGPDPIHH
Os04t0473800	OsFCP1	REVPTGPDPIHH
Zm00001eb07989	ZmFCP1	REVPTGPDPIHH
Bradi5g13241	BdFCP1	REVPTGPDPIHH
Sevir.7G142950v2	SvFCP1	REVPTGPDPIHH
TRITD2Av1G203550	TdFCP1a	REVPTGPDPIHH
TRITD2Bv1G167020	TdFCP1b	REVPTGPDPIHH

c CLV3 orthologs - CLE domain

Gene ID	Name	CLE domain sequence
AT2G27250	AtCLV3	RTVPSGPDPLHH
HORVU.MOREX.r3.4HG0360540	HvCLV3	RSVPAGPDPLHH
Os11g38270	OsFON2	RSVPAGPDPMHH
GRMZM2G372364	ZmCLE7	RAVPGGPDPLHH
Bradi4g14615	BdFON2	RMVPGGPDPLHH
Sevir.8G183800	SvFON2	RSVPGGPDPLHH

Supplementary Figure 5. **CLE peptides in barley and identification of FCP1 and CLV3 orthologs**

a List including all the barley CLE peptides identified by reciprocal BLAST using EnsemblePlants (https://plants.ensembl.org/index.html). **b** Identification of the barley FCP1 ortholog (HvFCP1), which shares an identical CLE domain sequence with the FCP1 peptide in *Oryza sativa japonica, Zea mays, Brachypodium distachyon, Setaria viridis* and *Triticum turgidum.* ***c*** Identification of the closest barley ortholog of the *Arabidopsis thaliana* CLV3 (HvCLV3), and comparison with CLV3 orthologs in *Oryza sativa japonica, Zea mays, Brachypodium distachyon, Setaria viridis* and *Triticum turgidum.* Gene ID, name and CLE domain amino acid sequence were added for each of the mentioned CLE peptides.

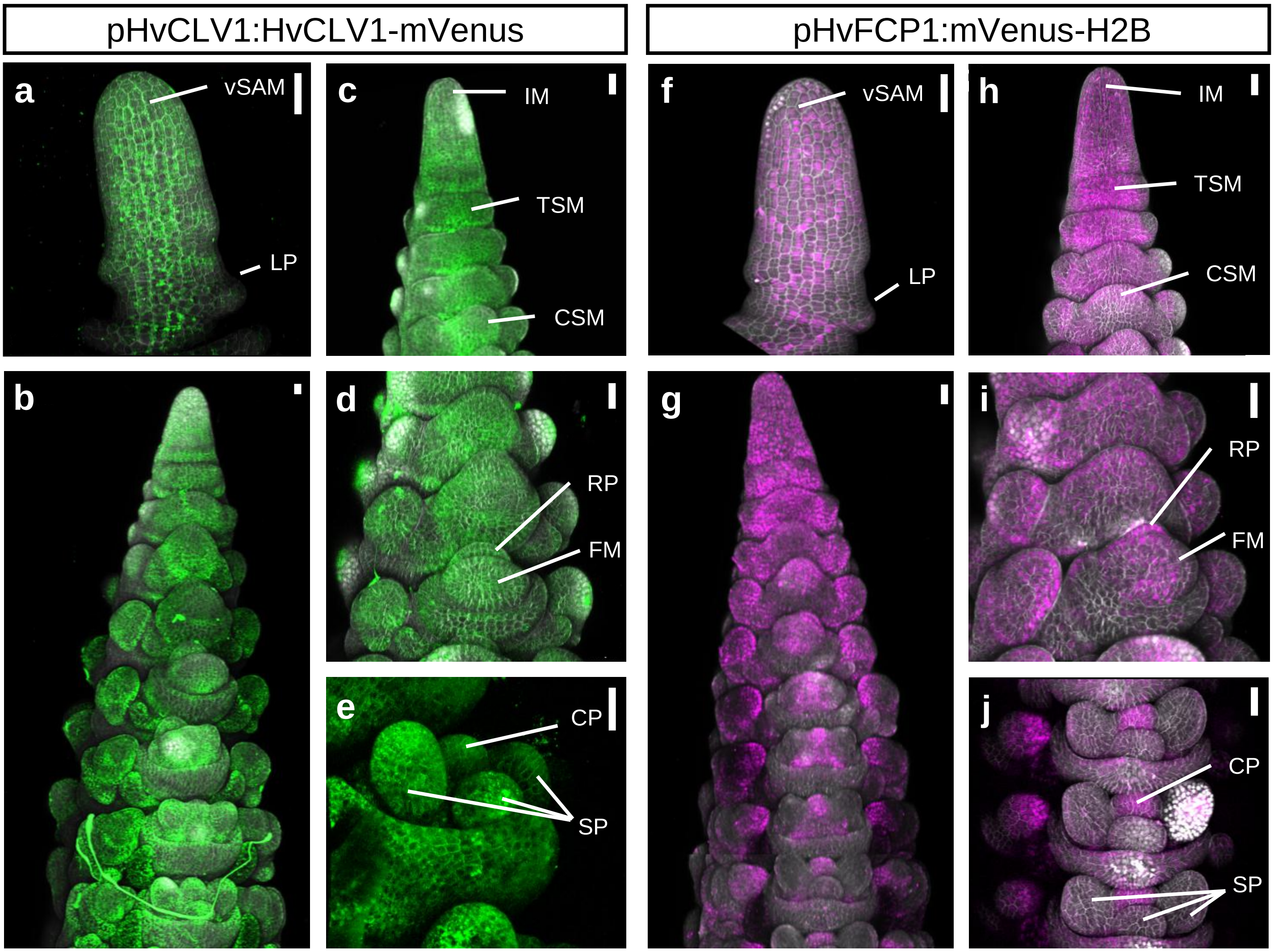

Supplementary Figure 6. **Comparison between HvCLV1 protein localisation and HvFCP1 promoter activity in barley inflorescence**

a-e HvCLV1 translational reporter line in barely inflorescence at W1 (a) and at W3.5 (b). Close-ups on HvCLV1 protein localisation in IM, TSM and CSM (c), RP and FM (d) and CP, SP (e). HvCLV1 proteins in green, DAPI-stained cell wall in grey. **f-j** *HvFCP1* transcriptional reporter line in barley inflorescence at W1 (f) and at W3.5 (g). Close-ups on *HvFCP1* expression in IM, TSM and CSM (h), RP and FM (i) and CP, SP (j). HvFCP1 expression in magenta, DAPI-stained cell wall in grey. Scalebar: 50 µm.

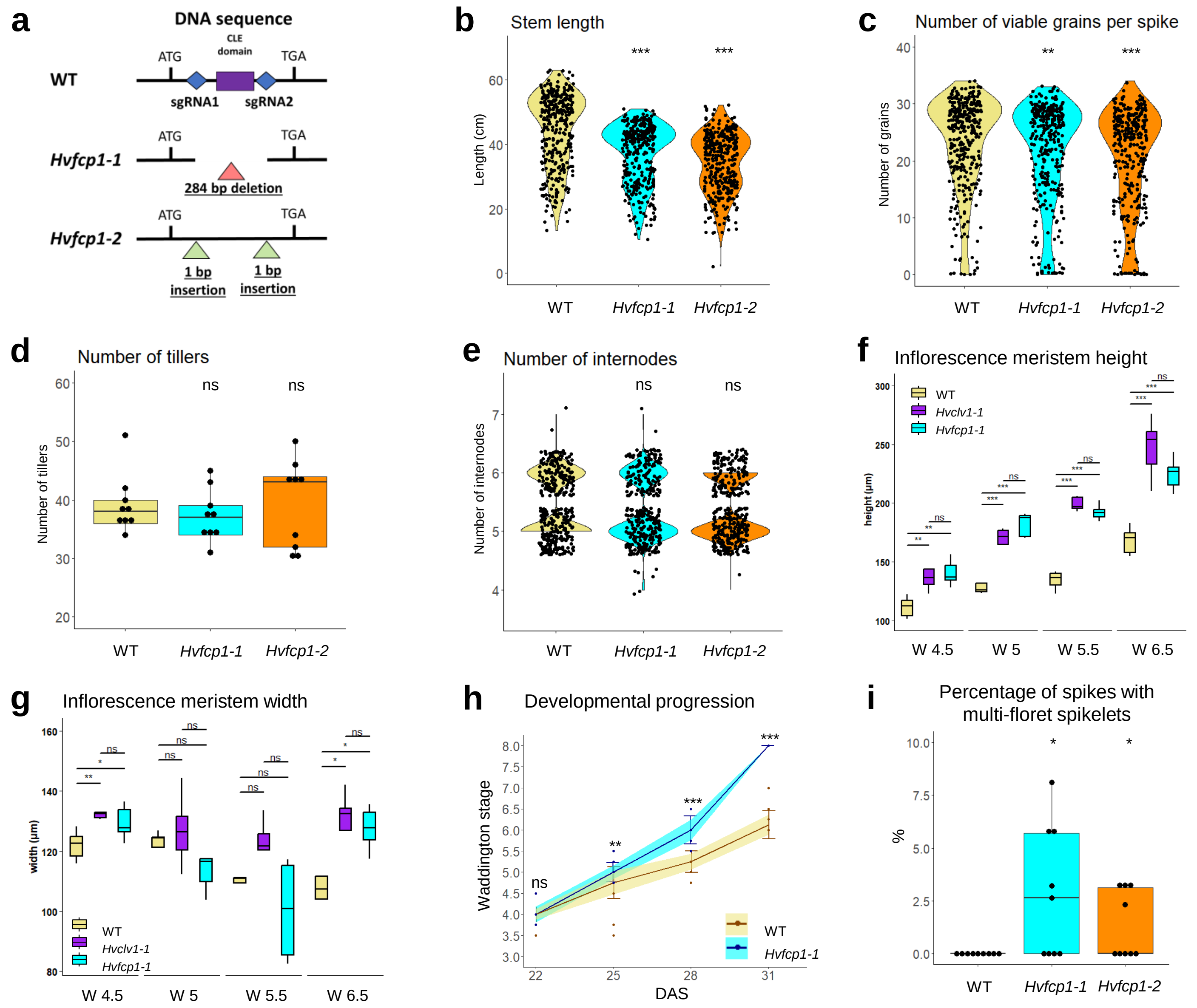

Supplementary Figure 7. **Phenotypes of *Hvfcp1* mutant alleles and IM shape**

a Schematic representation of the *HvFCP1* DNA sequence in WT and *Hvfcp1-1* and *Hvfcp1-2* mutant alleles. The blue squares indicate the regions targeted by the sgRNAs, the purple rectangle indicates the position of the HvFCP1 CLE domain, and triangles indicate the position of the selected mutation (insertions in green, deletions in red). **b-e** Plant phenotype: measurements of stem length (b), number of viable grains per spike (c), number of tillers (d), number of internodes (e). Dots indicate single measurement performed in n=9 mature plants over n=3 independent experiments and asterisks indicate the significant difference in comparison to WT. **f, g** Measurements of IM height and width at different W in WT (yellow), *Hvclv1-1* (purple) and *Hvfcp1-1* (cyan). IM width and height at different W were measured by tracing a horizontal line from the last visible spikelet primordium (IM width) and a perpendicular vertical line connecting it to the highest IM point (IM height) in WT (yellow) and *Hvfcp1-1* (cyan) inflorescences. Asterisks indicate the significant difference between genotypes coupled by the subtending horizontal line. **h** Developmental progression in WT (yellow) and *Hvfcp1-1* (cyan). Dots represent single measurements; error bars represent standard deviation, and the coloured ribbon is the interval of confidence. n=10 IMs per genotype for each W. **i** Percentage of spikes with multi-floret spikelets in WT plants and *Hvfcp1* mutant alleles. Dots represent the percentage per plant, and asterisks indicate the significant difference to WT. n=9 mature plants over n=3 independent experiment. Boxplots: median (center line); upper and lower quartiles (box limits); 1.5x interquartile range (whiskers); points, outliers. Statistics: asterisks indicate the significant difference in comparison to WT using a two-sided Pairwise Wilcoxon rank sum test (b,c,e,h,i) or a two-sided Pairwise t-test (d,f,g). ns = non-significant (p-value > 0.05); * (p-value < 0.05); ** (p-value < 0.01); *** (p-value < 0.001); **** (p-value < 0.0001).

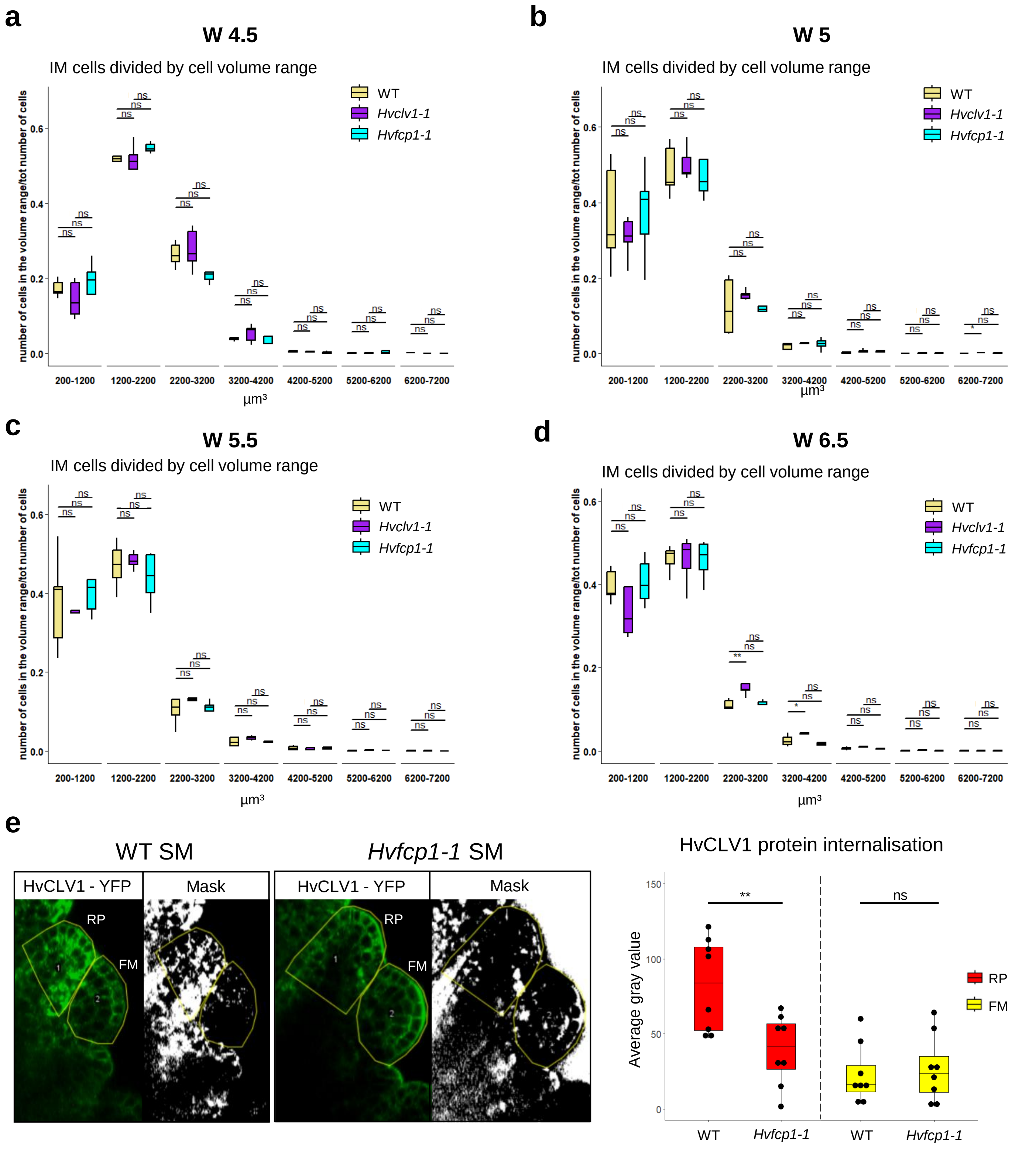

Supplementary Figure 8. **Inflorescence meristem Cell Volume Ratio and HvCLV1 internalisation in *Hvfcp1* RP and FM.**

a-d Quantifications of the Cell Volume Ratio (CVR) in WT (yellow), *Hvclv1-1* (purple) and *Hvfcp1-1* (cyan) in barley inflorescences at WS 4.5 (a), 5 (b), 6 (c) and 6.5 (d) respectively. In the y axis CVR (ratio between number of cells in a specific volume range and total number of cells of the IM), in the x axis cell volume ranges. n=5 IMs were analysed for each genotype and developmental stage over three independent experiments **e** Quantification of HvCLV1 protein internalisation in WT and *Hvfcp1* RP (red) and FM (yellow). n=8 central spikelets per genotype. Asterisks indicate the significant difference between genotypes coupled by the subtending horizontal line asterisks using a two-sided Pairwise t-test. Boxplots: median (center line); upper and lower quartiles (box limits); 1.5x interquartile range (whiskers); points, outliers. Statistics: ns = non-significant (p-value > 0.05); * (p-value < 0.05); ** (p-value < 0.01); *** (p-value < 0.001); **** (p-value < 0.0001).

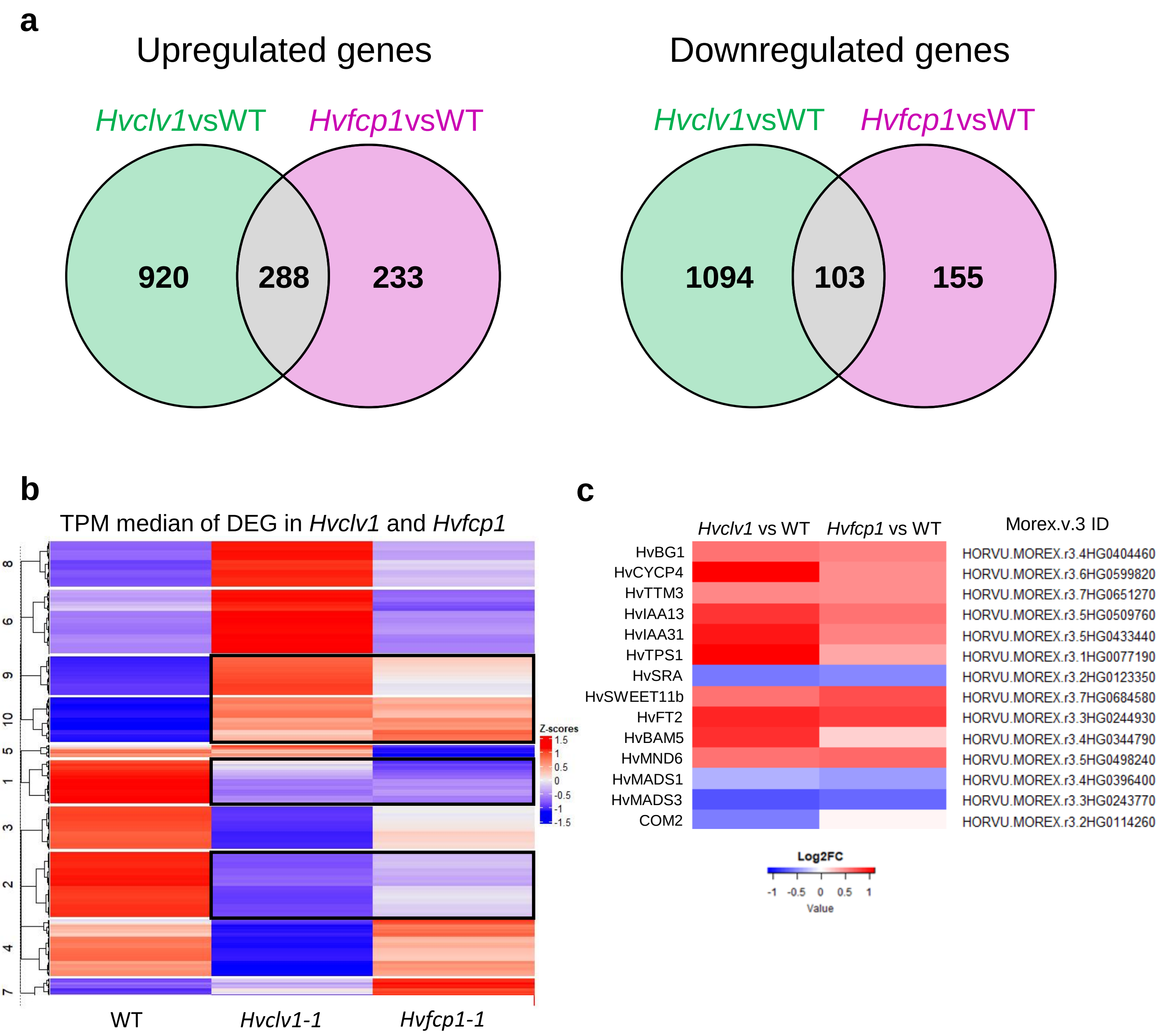

Supplementary Figure 9. **RNA sequencing in *Hvclv1* vs WT and *Hvfcp1* vs WT revealed a common gene regulatory network**

a Venn diagrams illustrating the number of upregulated ($Log_2FC > 0.5$) and downregulated ($Log_2FC < -0.5$) genes in *Hvclv1* vs WT (green) and *Hvfcp1* vs WT (magenta). Similarly regulated genes in grey. **b** Heatmap illustrating the z-score of median transcripts per million (TPM) values for each of the differently expressed genes (DEG) in *Hvclv1* vs WT and *Hvfcp1* vs WT. Clusters on the y-axis group genes with a similar expression trend between genotypes. Black rectangles highlight genes that are similarly regulated in *Hvclv1-1* and *Hvfcp1-1*. **c** Heatmap displaying gene name, Log_2FC values and Morex.v3 ID of mentioned DEG in *Hvclv1* vs WT and *Hvfcp1* vs WT.

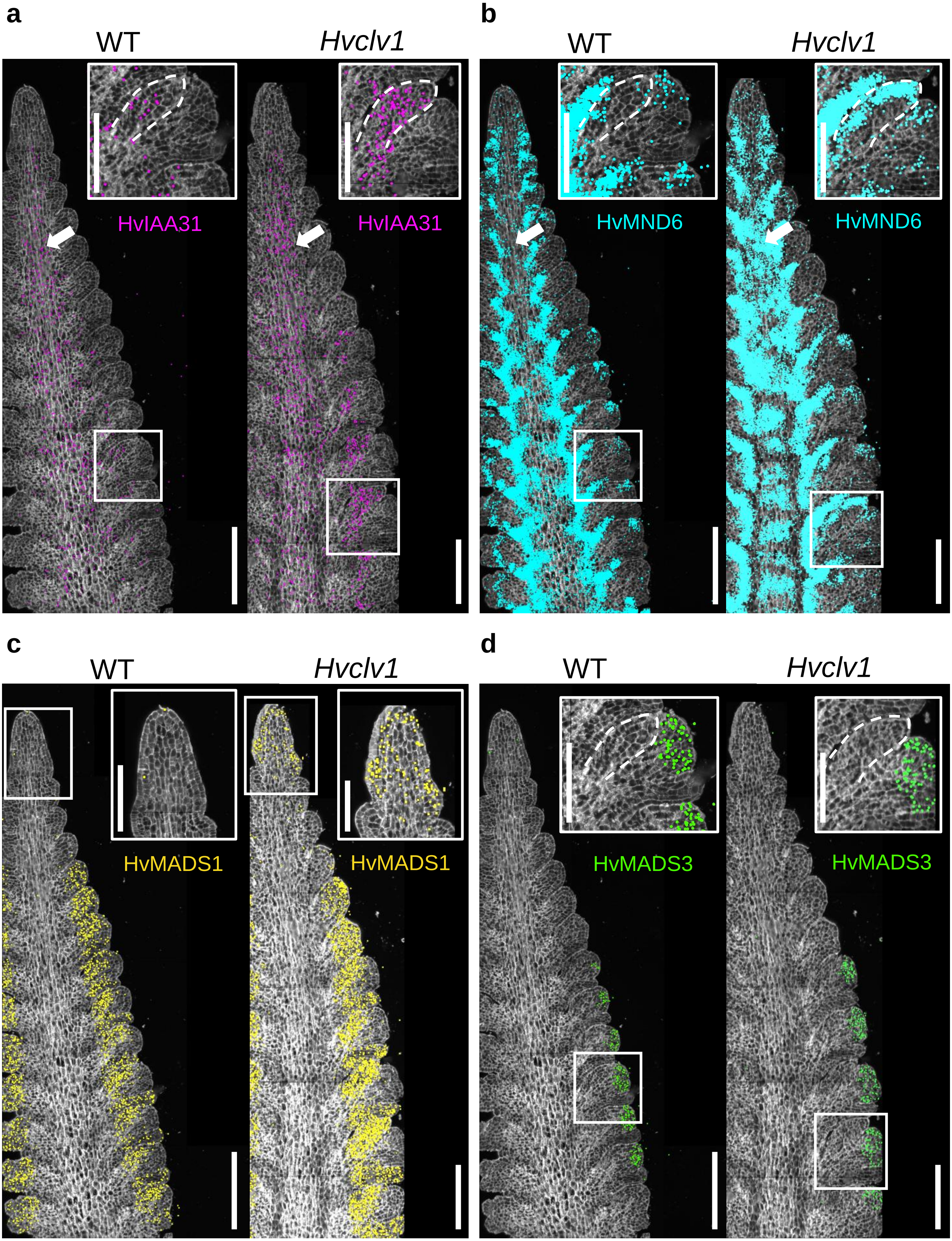
a
WT
Hvclv1
HvIAA31
HvIAA31
b
WT
Hvclv1
HvMND6
HvMND6
c
WT
Hvclv1
HvMADS1
HvMADS1
d
WT
Hvclv1
HvMADS3
HvMADS3

Supplementary Figure 10. **Detailed expression of some differentially expressed genes in WT and *Hvclv1* inflorescences**

a-d smRNA-FISH results showing the localisation of *HvIAA31* (a), *HvMADS6* (b), *HvMADS1* (c), and *HvMADS3* (d) transcripts in WT and *Hvclv1-1* inflorescences at W3.5. White arrows indicate the main rachis and white boxes indicate the region displayed in the zoom-in boxes. The RP is marked with a white segmented line. Scale bars: 100 μm (inflorescence sections) and 50 μm (zoom-in boxes).

Supplementary Table 1. **Primers used in this study**

Construct	Aim	Primer	Primer sequence
pHvCLV1:HvCLV1-mVenus	amplifying promoter	GK-HvpCLV1-fw-AscI	AAAGGCGCGCCGTTTATTTATTGAAGTATTAATCA
		GK-HvpCLV1-rv3	GCAGGTGAGGTGGCGGCATTGTG
	amplifying CDS	GK-HvCLV1-fw+CACC	CACCATGCCGCCACCTCACCTGC
		GK-HvCLV1-rv-stop2	GAAGGAGAGGATGAGGTCGTCGTCGG
pHvFCP1:mVenus-H2B	amplifying promoter	GK-HvpCLE402-fw+CACC	CACCCATGCGACGTTCCCCAACAGCCT
		GK-HvpCLE402-rv	CCAATCCGGCCTTGGCCCTAGCG
p35sHyg-Cas9_HvCLV1	sgRNA	HvCLV1_sgRNA_Fw	agcaGGCCCCTTCCTCCGGCTCCC
		HvCLV1_sgRNA_Rv	aaacGGGAGCCGGAGGAAGGGGCC
p35sHyg-Cas9_HvFCP1	sgRNA_1	HvFCP1_sgRNA1_Fw	agcaGCAGGACCTGCAGGAGAAGC
		HvFCP1_sgRNA1_Rv	aaacGCTTCTCCTGCAGGTCCTGC
	sgRNA_2	HvFCP1_sgRNA2_Fw	agcaCAGGGCGACTGCCGGCGCCT
		HvFCP1_sgRNA2_Rv	aaacAGGCGCCGGCAGTCGCCCTG
p35sHyg-Cas9_HvCLV1	mutant selection by genotyping	HvCLV1_gene_Fw	CGTGCCACTCACATCACATC
		HvCLV1_gene_Rv	TGGTGAGGTTGGTTAGGGAGT
p35sHyg-Cas9_HvFCP1	mutant selection by genotyping	HvFCP1_gene_Fw	CATGCGTTCGTTGCTCTCTA
		HvFCP1_gene_Rv	CCTCAGAATGGACCCAACAC
	Selection of Cas9-free plants	Fl_Cas9_Fw	TTGATGTGGGTTTTACTGATGC
		Fl_Cas9_Rv	CTTGTAGCCTCGGCTGTCTC
		Fl_Hyg_Fw	ATTTCGGCTCCAACAATGTC
		Fl_Hyg_Rv	GCAGGTCACTGGATTTTGGT

Supplementary Notes 1: ***Hvclv1* mutant alleles**

HvCLV1 (HORVU.MOREX.r3.7HG0747230) Location: Chr7H 618,787,699 - 618,791,569

Hvclv1-1 carries a 1bp insertion after 70bp that caused a shift in the reading frame and an early stop codon after 476 amino acids (aa), generating a misfolded protein which only shares the first 23aa with the WT sequence (1016aa). *Hvclv1-2* carries a 29bp deletion 41bp after the coding start that caused a shift in the reading frame and an early stop codon after 466 aa. *Hvclv1-3* carries a 21 bp deletion after 63bp from the coding start, which allowed the formation of a protein almost identical to the WT apart from 7 missing amino acids (SGSPDRD) in position 22 to 28 in the N-terminal region that disrupt the signal peptide sequence.

<u>Alignment: sgRNA target sequence (blue), insertion (green), deletion (red)</u>

```
HvCLV1          ATGCCGCCACCTCACCTGCTCACCATCCTCCTACCTCTCCTCCTCCTCCTCCCGGCCCCT
Hvclv1-1        ATGCCGCCACCTCACCTGCTCACCATCCTCCTACCTCTCCTCCTCCTCCTCCCGGCCCCT
Hvclv1-2        ATGCCGCCACCTCACCTGCTCACCATCCTCCTACCTCTCCTCC-----------------
Hvclv1-3        ATGCCGCCACCTCACCTGCTCACCATCCTCCTACCTCTCCTCCTCCTCCTCCCGGCCCCT

                *******************************************

HvCLV1          TCCTCCGGCT-CCCCGGACCGCGACATCTACGCGCTCGCCAAGATCAAGGCCGCCCT…
Hvclv1-1        TCCTCCGGCTTCCCCGGACCGCGACATCTACGCGCTCGCCAAGATCAAGGCCGCCCT…
Hvclv1-2        -------------CCGGACCGCGACATCTACGCGCTCGCCAAGATCAAGGCCGCCCT…
Hvclv1-3        TCC---------------------CATCTACGCGCTCGCCAAGATCAAGGCCGCCCT…

                                        *********************************
```

<u>Signal peptide and cleavage position prediction (PredSi)</u>

**Matrix:**	Eukarya
**Truncation:**	70 residues
**Cleavage position:**	23
**Score:**	0.9205
**Secreted protein:**	predicted for secretion

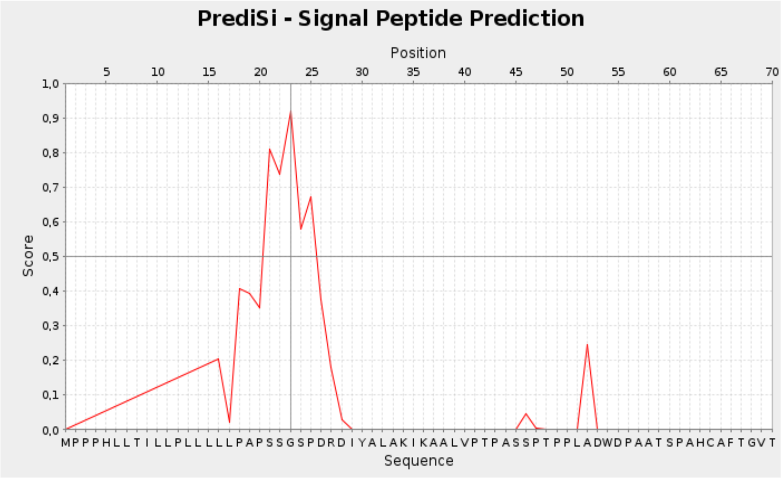

HvCLV1:

```
MPPPHLLTILLPLLLLLPAPSSGSPDRDIYALAKIKAALVPTPASSPTPPLADWDPAATSPAHCAFTGVT
|   |    |    |    |    |    |    |    |    |    |    |    |    |    |
1        10        20        30        40        50        60        70
```

HvCLV1 WT protein sequence

MPPPHLLTILLPLLLLLPAPSSGSPDRDIYALAKIKAALVPTPASSPTPPLADWDPAATSPAHCAFTGVTCDAATSR
VVAINLTALPLHAGTLPPELALLDSLTNLTIAACSLPGRVPAGLPSLPSLRHLNLSNNNLSGPFPAGDGQTTLYFPS
IEVLDCYNNNLSGPLPPFGAAHKAALRYLHLGGNYFSGPIPVAYGDVASLEYLGLNGNALSGRIPPDLARLGRLRSL
YVGYFNQYDGGVPPEFGGLRSLVLLDMSSCNLTGPIPPELGKLKNLDTLFLLWNRLSGEIPPELGELQSLQLLDLSV
NDLAGEIPATLAKLTNLRLLNLFRNHLRGGIPGFVADLPDLEVLQLWENNLTGSLPPGLGRNGRLRNLDVTTNHLTG
TVPPDLCAGGRLEMLVLMDNAFFGPIPESLGACKTLVRVRLSKNFLSGAVPAGLFDLPQANMLELTDNLLTGGLPDV
IGGGKIGMLLLGNNGIGGRIPPAIGNLPALQTLSLESNNFTGELPPEIGRLRNLSRLNVSGNHLTGAIPEELTRCSS
LAAVDVSRNRLTGVIPESITSLKILCTLNVSRNALSGELPTEMSNMTSLTTLDVSYNALTGDVPMQGQFLVFNESSF
VGNPGLCGGPLTGSSNDDACSSSSNHGGGGVLSLRRWDSKKMLVCLAAVFVSLVAAFLGGRKGCEAWREAARRRSGA
WKMTVFQQRPGFSADDVVECLQEDNIIGKGGAGIVYHGVTRGGGAELAIKRLVGRGVGGDRGFSAEVGTLGRIRHRN
IVRLLGFVSNRETNLLLYEYMPNGSLGEMLHGGKGGHLGWDARARVALEAARGLCYLHHDCAPRIIHRDVKSNNILL
DSAFEAHVADFGLAKFLGGAAGASECMSAIAGSYGYIAPEYAYTLRVDEKSDVYSFGVVLLELITGRRPVGGFGDGV
DIVHWVRKATAELPDTAAAVLAVADCRLSPEPVPLLVGLYDVAMACVEEASTDRPTMREVVHMLSQPALVAPTAVVD
ENTARPDDDLILSF*

Hvclv1 mutant alleles protein sequence

Hvclv1-1

MPPPHLLTILLPLLLLLPAPSSGFPGPRHLRARQDQGRPRAHPRILPDAAARRLGPGGDIPSPLRIHRRHMRRRHLP
RRRHQPHRPPAPRRHAAPGARPPRLPNQPHHRRLLPPRPRPRGPPVPAIPPPPQPLQQQPLRPLPRRRRTDNVVLPV
HRGPRLLQQQPLRPAPALRRRAQGRAPLPPPRRELLLRPHPGGLRRRRQPRVPRPQRQRALRQDPAGPGPAGPAPEP
LRRLLQPVRRRRAARVRRAAQPRAARHEQLQPHRPHPARARQAQEPRHALPPLEPIVWRDSARAGGAPEPPVAGPVR
QRPRRRDTGDPGQAHEPQAAQPVPEPPPRRDTRVRRRPAGPRGAAALGEQPHRQPPAGTRAQRPAQEPRRHHQPPHR
HRAAGPLRGREARDARAHGQRLLRPHPGVAGRVQDAGARPPQQELPQRRRAGRALRPAAGQHARAHRQPAHGRPPRR
DRRRQDRHAAAGE*

Hvclv1-2

MPPPHLLTILLPLLPGPRHLRARQDQGRPRAHPRILPDAAARRLGPGGDIPSPLRIHRRHMRRRHLPRRRHQPHRPP
APRRHAAPGARPPRLPNQPHHRRLLPPRPRPRGPPVPAIPPPPQPLQQQPLRPLPRRRRTDNVVLPVHRGPRLLQQQ
PLRPAPALRRRAQGRAPLPPPRRELLLRPHPGGLRRRRQPRVPRPQRQRALRQDPAGPGPAGPAPEPLRRLLQPVRR
RRAARVRRAAQPRAARHEQLQPHRPHPARARQAQEPRHALPPLEPIVWRDSARAGGAPEPPVAGPVRQRPRRRDTGD
PGQAHEPQAAQPVPEPPPRRDTRVRRRPAGPRGAAALGEQPHRQPPAGTRAQRPAQEPRRHHQPPHRHRAAGPLRGR
EARDARAHGQRLLRPHPGVAGRVQDAGARPPQQELPQRRRAGRALRPAAGQHARAHRQPAHGRPPRRDRRRQDRHAA
AGE*

Hvclv1-3

MPPPHLLTILLPLLLLLPAPSIYALAKIKAALVPTPASSPTPPLADWDPAATSPAHCAFTGVTCDAATSRVVAINLT
ALPLHAGTLPPELALLDSLTNLTIAACSLPGRVPAGLPSLPSLRHLNLSNNNLSGPFPAGDGQTTLYFPSIEVLDCY
NNNLSGPLPPFGAAHKAALRYLHLGGNYFSGPIPVAYGDVASLEYLGLNGNALSGRIPPDLARLGRLRSLYVGYFNQ
YDGGVPPEFGGLRSLVLLDMSSCNLTGPIPPELGKLKNLDTLFLLWNRLSGEIPPELGELQSLQLLDLSVNDLAGEI
PATLAKLTNLRLLNLFRNHLRGGIPGFVADLPDLEVLQLWENNLTGSLPPGLGRNGRLRNLDVTTNHLTGTVPPDLC
AGGRLEMLVLMDNAFFGPIPESLGACKTLVRVRLSKNFLSGAVPAGLFDLPQANMLELTDNLLTGGLPDVIGGGKIG
MLLLGNNGIGGRIPPAIGNLPALQTLSLESNNFTGELPPEIGRLRNLSRLNVSGNHLTGAIPEELTRCSSLAAVDVS
RNRLTGVIPESITSLKILCTLNVSRNALSGELPTEMSNMTSLTTLDVSYNALTGDVPMQGQFLVFNESSFVGNPGLC
GGPLTGSSNDDACSSSSNHGGGGVLSLRRWDSKKMLVCLAAVFVSLVAAFLGGRKGCEAWREAARRRSGAWKMTVFQ
QRPGFSADDVVECLQEDNIIGKGGAGIVYHGVTRGGGAELAIKRLVGRGVGGDRGFSAEVGTLGRIRHRNIVRLLGF
VSNRETNLLLYEYMPNGSLGEMLHGGKGGHLGWDARARVALEAARGLCYLHHDCAPRIIHRDVKSNNILLDSAFEAH
VADFGLAKFLGGAAGASECMSAIAGSYGYIAPEYAYTLRVDEKSDVYSFGVVLLELITGRRPVGGFGDGVDIVHWVR
KATAELPDTAAAVLAVADCRLSPEPVPLLVGLYDVAMACVEEASTDRPTMREVVHMLSQPALVAPTAVVDENTARPD
DDLILSF*

Supplementary Notes 2: ***Hvfcp1* mutant alleles**

HvFCP1 (HORVU.MOREX.r3.2HG0174890) Location: Chr2H 523,068,208 - 523,069,031

We generated two independent knock-out mutant alleles by CRISPR-Cas9, called *Hvfcp1-1* and *Hvfcp1-2*. The 284bp deletion in *Hvfcp1-1* removed part of the first exon and the entire second exon, which normally carries the conserved CLE domain. *Hvfcp1-2*, carries two 1bp insertions at the +298bp and +543bp positions. The first insertion caused a shift in the reading frame that altered the entire amino acid sequence of the predicted peptide.

<u>sgRNAs target sequences (blue), insertion (green), deletion (red)</u>

```
HvFCP1          ATGGCTCATGCCGCCGACGCGAGGTCGCGCTGCGTCGTCGCGGTGCTCTTCGCCGTAGCC
Hvfcp1-1        ATGGCTCATGCCGCCGACGCGAGGTCGCGCTGCGTCGTCGCGGTGCTCTTCGCCGTAGCC
Hvfcp1-2        ATGGCTCATGCCGCCGACGCGAGGTCGCGCTGCGTCGTCGCGGTGCTCTTCGCCGTAGCC
                ************************************************************

HvFCP1          GTCTTCCTCGCCTGCTTGCCGCCCGCCGCCGCCTCCTCCTCGTCTTCCCGGGCAGGTACG
Hvfcp1-1        GTCTTCCTCGCCTGCTTGCCGCCCGCCGCCGCCTCCTCCTCGTCTTCCCGGGCAGGTACG
Hvfcp1-2        GTCTTCCTCGCCTGCTTGCCGCCCGCCGCCGCCTCCTCCTCGTCTTCCCGGGCAGGTACG
                ************************************************************

HvFCP1          TGCGTCGTCCCGTCCGCCATGCGTTCGTTGCTCTCTACAACCCCCGCCGCAAGGCCACCT
Hvfcp1-1        TGCGTCGTCCCGTCCGCCATGCGTTCGTTGCTCTCTACAACCCCCGCCGCAAGGCCACCT
Hvfcp1-2        TGCGTCGTCCCGTCCGCCATGCGTTCGTTGCTCTCTACAACCCCCGCCGCAAGGCCACCT
                ************************************************************

HvFCP1          CCCTGGTTCTCGCGCCGCACGGGAATCTCCTGCGCTCTTTGACGCCTTTGTTGGTCATCT
Hvfcp1-1        CCCTGGTTCTCGCGCCGCACGGGAATCTCCTGCGCTCTTTGACGCCTTTGTTGGTCATCT
Hvfcp1-2        CCCTGGTTCTCGCGCCGCACGGGAATCTCCTGCGCTCTTTGACGCCTTTGTTGGTCATCT
                ************************************************************

HvFCP1          CCCTCGCAGCGGCGGCGGCATTGCAACGAGTCGAGATGGCGGCCATGTACACCCCGC
Hvfcp1-1        CCCTCGCAGCGGCGGCGGCATTGCAACGAGTCGAGAT-----------------------
Hvfcp1-2        CCCTCGCAGCGGCGGCGGCATTGCAACGAGTCGAGATGGCGGCCATGTACACCCCGC
                *************************************

HvFCP1          AGGACCTGCAGGAGAAG-CCGGATGTGACCAAGGTACGTACGCGGCCGCCATGTTACGGC
Hvfcp1-1        ------------------------------------------------------------
Hvfcp1-2        AGGACCTGCAGGAGAAAGCCGGATGTGACCAAGGTACGTACGCGGCCGCCATGTTACGGC

HvFCP1          TTCGGGCCGAAGGAAAGGCGGCTCCTTTGGTGGTTTCTTGCTGTCGTGTTTCGAGCTCAT
Hvfcp1-1        ------------------------------------------------------------
Hvfcp1-2        TTCGGGCCGAAGGAAAGGCGGCTCCTTTGGTGGTTTCTTGCTGTCGTGTTTCGAGCTCAT

HvFCP1          GGGGTTTTGATTTTCGATGCGCAGGACGCGGAGGAGGACGTGAGCACGACGGGGTTCGGC
Hvfcp1-1        ------------------------------------------------------------
Hvfcp1-2        GGGGTTTTGATTTTCGATGCGCAGGACGCGGAGGAGGACGTGAGCACGACGGGGTTCGGC

HvFCP1          GCGGAGGAGGAGAGGGAGGTGCCCACCGGGCCGGACCCCATCCACCACCACGGCAGGGGA
Hvfcp1-1        ------------------------------------------------------------
Hvfcp1-2        GCGGAGGAGGAGAGGGAGGTGCCCACCGGGCCGGACCCCATCCACCACCACGGCAGGGGA

HvFCP1          CCCAGG-CGCCGGCAGTCGCCCTGATCGCGCGGCAGGTGGAGGATGCTTCCGTGGGTCG…
Hvfcp1-1        --------------------------CGCGCGGCAGGTGGAGGATGCTTCCGTGGGTCG…
Hvfcp1-2        CCCAGGGCGCCGGCAGTCGCCCTGATCGCGCGGCAGGTGGAGGATGCTTCCGTGGGTCG…
                                          *********************************
```

WT protein sequence (CLE domain highlighted in yellow)

MAHAADARSRCVVAVLFAVAVFLACLPPAAASSSSSRAAAAAALQRVEMAAMYTPQDLQE

KPDVTKDAEEDVSTTGFGAEEEREVPTGPDPIHHHGRGPRRRQSP*

Hvfcp1 mutant alleles protein sequence

Hvfcp1-1

MAHAADARSRCVVAVLFAVAVFLACLPPAAASSSSSRAGTCVVPSAMRSLLSTTPAARPPPWFSRRTGISCAL*

Hvfcp1-2

MAHAADARSRCVVAVLFAVAVFLACLPPAAASSSSSRAAAAAALQRVEMAAMYTPQDLQEKAGCDQGRGGGREHDGV
RRGGGEGGAHRAGPHPPPRQGTQGAGSRPDRAAGGGCFRGSVHPA*

Alignment

```
HvFCP1          MAHAADARSRCVVAVLFAVAVFLACLPPAAASSSSSRAAAAAALQRVEMAAMYTPQDLQE
Hvfcp1-2        MAHAADARSRCVVAVLFAVAVFLACLPPAAASSSSSRAAAAAALQRVEMAAMYTPQDLQE
Hvfcp1-1        MAHAADARSRCVVAVLFAVAVFLACLPPAAASSSSSRAGTCVVPSAMRSLLSTTPAAR--
                **************************************.:... . :.     **

HvFCP1          KP--DVTKDAEEDVSTTGFGAEEEREVPTGPDPIHHHGRGPRRRQSP-------------
Hvfcp1-2        KAGCDQGRGGGREHDGVRRGGGEGGAHRAGPHPPPRQGTQGAGSRPDRAAGGGCFRGSVH
Hvfcp1-1        ------------------------------PPPWFSRRTGISCAL---------------
                                              * *   :

HvFCP1          --
Hvfcp1-2        PA
Hvfcp1-1        --
```
