# Supplementary material for: CLAVATA signalling shapes barley inflorescence by controlling activity and determinacy of shoot meristem and rachilla
Source: Nat Commun. 2025 Apr 26;16:3937. doi: 10.1038/s41467-025-59330-z (PMC12033307; doi:10.1038/s41467-025-59330-z)
Supplement: Supplementary file 2 — Description of Additional Supplementary Files [file 41467_2025_59330_MOESM2_ESM.pdf]

Description of Additional Supplementary Files:

Supplementary Data 1: Protein kinase domain containing proteins phylogenetic tree
Maximum likelihood phylogenetic tree displaying all protein kinase domain containing proteins in the selected species (*Oryza sativa, Triticum turgidum, Zea mays, Hordeum vulgare, Arabidopsis thaliana and Solanum lycopersicum*). The displayed bootstrap values for each node are based on 1000 bootstrap replicates.

Supplementary Data 2: Differentially expressed genes in *Hvclv1-1* and *Hvfcp1-1* versus WT
Dataset displaying all the significant differentially expressed genes (DEG) in *Hvclv1* and *Hvfcp1* compared to WT (FDR<0.05 and $log_2FC \leq -0.5$ or $log_2FC \geq 0.5$). For each DEG the Morex.v3 gene ID and description are provided, in addition to the closest ortholog in *Arabidopsis thaliana* and *Oryza sativa*, mean TPM values, and Log_2Fold Change values (Log_2FC) of gene expression in mutants compared to WT.
