# Supplementary material for: CLAVATA signalling shapes barley inflorescence by controlling activity and determinacy of shoot meristem and rachilla
Source: Nat Commun. 2025 Apr 26;16:3937. doi: 10.1038/s41467-025-59330-z (PMC12033307; doi:10.1038/s41467-025-59330-z)
Supplement: Supplementary file 5 — Reporting Summary [file 41467_2025_59330_MOESM5_ESM.pdf]

nature portfolio

Corresponding author(s): Rüdiger Simon

Last updated by author(s): Feb 24, 2025

Reporting Summary

Nature Portfolio wishes to improve the reproducibility of the work that we publish. This form provides structure for consistency and transparency in reporting. For further information on Nature Portfolio policies, see our Editorial Policies and the Editorial Policy Checklist.

Statistics

For all statistical analyses, confirm that the following items are present in the figure legend, table legend, main text, or Methods section.

n/a	Confirmed	
☐	☒	The exact sample size (*n*) for each experimental group/condition, given as a discrete number and unit of measurement
☐	☒	A statement on whether measurements were taken from distinct samples or whether the same sample was measured repeatedly
☐	☒	The statistical test(s) used AND whether they are one- or two-sided<br>*Only common tests should be described solely by name; describe more complex techniques in the Methods section.*
☒	☐	A description of all covariates tested
☐	☒	A description of any assumptions or corrections, such as tests of normality and adjustment for multiple comparisons
☐	☒	A full description of the statistical parameters including central tendency (e.g. means) or other basic estimates (e.g. regression coefficient) AND variation (e.g. standard deviation) or associated estimates of uncertainty (e.g. confidence intervals)
☒	☐	For null hypothesis testing, the test statistic (e.g. *F*, *t*, *r*) with confidence intervals, effect sizes, degrees of freedom and *P* value noted<br>*Give P values as exact values whenever suitable.*
☒	☐	For Bayesian analysis, information on the choice of priors and Markov chain Monte Carlo settings
☒	☐	For hierarchical and complex designs, identification of the appropriate level for tests and full reporting of outcomes
☒	☐	Estimates of effect sizes (e.g. Cohen's *d*, Pearson's *r*), indicating how they were calculated

Our web collection on statistics for biologists contains articles on many of the points above.

Software and code

Policy information about availability of computer code

Data collection	We used Zen software to collect microscopy images.
Data analysis	Microscopy images were analysed using the software Fiji. All the statistical tests were performed using the packages stats and rstatix in R Studio (RStudio Team 2022). The software HMMER (V 3.2) was used for phylogenetic analysis. The 3D reconstruction of inflorescence meristems was performed using MorphoGraphX 2.0. Analysis of RNA sequencing data was performed using: Salmon (v. 0.14.1) and the R packages: EdgeR (v3.32.1) and ThreeDRNAseq.

For manuscripts utilizing custom algorithms or software that are central to the research but not yet described in published literature, software must be made available to editors and reviewers. We strongly encourage code deposition in a community repository (e.g. GitHub). See the Nature Portfolio guidelines for submitting code & software for further information.

Data

Policy information about availability of data

All manuscripts must include a data availability statement. This statement should provide the following information, where applicable:

- Accession codes, unique identifiers, or web links for publicly available datasets
- A description of any restrictions on data availability
- For clinical datasets or third party data, please ensure that the statement adheres to our policy

Source data for plots in Figures 1, 2, 4, 5 and Supplementary Figures 2, 4, 7, 8 are provided with this paper in Source data file. All the microscopy images generated for this study were deposited in BioImage Archive (https://www.ebi.ac.uk/biostudies/bioimages/studies/S-BIAD1800; accession number: S-BIAD1800). The FASTQ

files from RNA sequencing in WT, Hvclv1-1 and Hvfcp1-1 inflorescences have been deposited in the Sequence Read Archive (SRA) repository (http://www.ncbi.nlm.nih.gov/bioproject/1245663; BioProject: PRJNA1245663). FASTQ files from RNA-sequencing were deposited under BioSample accessions: SAMN47761230, SAMN47761231, SAMN47761232, SAMN47761233, SAMN47761234, SAMN47761235, SAMN47761236, SAMN47761237, SAMN47761238.

Research involving human participants, their data, or biological material

Policy information about studies with human participants or human data. See also policy information about sex, gender (identity/presentation), and sexual orientation and race, ethnicity and racism.

Reporting on sex and gender	*Use the terms sex (biological attribute) and gender (shaped by social and cultural circumstances) carefully in order to avoid confusing both terms. Indicate if findings apply to only one sex or gender; describe whether sex and gender were considered in study design; whether sex and/or gender was determined based on self-reporting or assigned and methods used. Provide in the source data disaggregated sex and gender data, where this information has been collected, and if consent has been obtained for sharing of individual-level data; provide overall numbers in this Reporting Summary. Please state if this information has not been collected. Report sex- and gender-based analyses where performed, justify reasons for lack of sex- and gender-based analysis.*
Reporting on race, ethnicity, or other socially relevant groupings	*Please specify the socially constructed or socially relevant categorization variable(s) used in your manuscript and explain why they were used. Please note that such variables should not be used as proxies for other socially constructed/relevant variables (for example, race or ethnicity should not be used as a proxy for socioeconomic status). Provide clear definitions of the relevant terms used, how they were provided (by the participants/respondents, the researchers, or third parties), and the method(s) used to classify people into the different categories (e.g. self-report, census or administrative data, social media data, etc.) Please provide details about how you controlled for confounding variables in your analyses.*
Population characteristics	*Describe the covariate-relevant population characteristics of the human research participants (e.g. age, genotypic information, past and current diagnosis and treatment categories). If you filled out the behavioural & social sciences study design questions and have nothing to add here, write "See above."*
Recruitment	*Describe how participants were recruited. Outline any potential self-selection bias or other biases that may be present and how these are likely to impact results.*
Ethics oversight	*Identify the organization(s) that approved the study protocol.*

Note that full information on the approval of the study protocol must also be provided in the manuscript.

Field-specific reporting

Please select the one below that is the best fit for your research. If you are not sure, read the appropriate sections before making your selection.

[x] Life sciences [ ] Behavioural & social sciences [ ] Ecological, evolutionary & environmental sciences

For a reference copy of the document with all sections, see nature.com/documents/nr-reporting-summary-flat.pdf

Life sciences study design

All studies must disclose on these points even when the disclosure is negative.

Sample size	All plant phenotypes were evaluated by measuring all tillers from three plants across three independent experiments conducted under controlled greenhouse conditions. For time-course microscopy analysis of barley inflorescence phenotypes, plants were grown in 96-well trays within a climate chamber, with 5 to 10 replicates measured at each developmental stage (the exact sample size is mentioned in the figure legends). This sample size was deemed sufficient to demonstrate significant differences in inflorescence phenotypes across multiple time points.
Data exclusions	No data were excluded.
Replication	All attempts at replication were successful.
Randomization	Plants were always grouped by genotype and grown under the same conditions.
Blinding	Most of the experiments were not blind because the results are quantitative and not subjective. Blinding was not feasible in most of the experiments, while a blind analysis was performed for results in Figure 4 A.

Reporting for specific materials, systems and methods

We require information from authors about some types of materials, experimental systems and methods used in many studies. Here, indicate whether each material, system or method listed is relevant to your study. If you are not sure if a list item applies to your research, read the appropriate section before selecting a response.

Materials & experimental systems

n/a	Involved in the study
☒	☐ Antibodies
☒	☐ Eukaryotic cell lines
☒	☐ Palaeontology and archaeology
☒	☐ Animals and other organisms
☒	☐ Clinical data
☒	☐ Dual use research of concern
☐	☒ Plants

Methods

n/a	Involved in the study
☒	☐ ChIP-seq
☒	☐ Flow cytometry
☒	☐ MRI-based neuroimaging

Dual use research of concern

Policy information about dual use research of concern

Hazards

Could the accidental, deliberate or reckless misuse of agents or technologies generated in the work, or the application of information presented in the manuscript, pose a threat to:

No	Yes
☒	☐ Public health
☒	☐ National security
☒	☐ Crops and/or livestock
☒	☐ Ecosystems
☒	☐ Any other significant area

Experiments of concern

Does the work involve any of these experiments of concern:

No	Yes
☒	☐ Demonstrate how to render a vaccine ineffective
☒	☐ Confer resistance to therapeutically useful antibiotics or antiviral agents
☒	☐ Enhance the virulence of a pathogen or render a nonpathogen virulent
☒	☐ Increase transmissibility of a pathogen
☒	☐ Alter the host range of a pathogen
☒	☐ Enable evasion of diagnostic/detection modalities
☒	☐ Enable the weaponization of a biological agent or toxin
☒	☐ Any other potentially harmful combination of experiments and agents

Plants

Seed stocks	Seed stocks of the barley cultivars Golden Promise and Golden Promise Fast were provided by Maria von Korff Schmising. A detailed description of the seed stock generation can be found in Gol, L., Haraldsson, E. B., & von Korff, M. (2021). Ppd-H1 integrates drought stress signals to control spike development and flowering time in barley. Journal of Experimental Botany, 72(1), 122–136.
Novel plant genotypes	A comprehensive description of the methods used to generate each novel genotype can be found in the Methods section under "Plasmid Construction and Plant Transformation."
Authentication	All genetically modified plants were screened by PCR, and the Cas9 sequence was eliminated through segregation and backcrossing. The absence of Cas9 was confirmed over at least three consecutive generations using PCR and resistance screening.
